# The Development and Evaluation of the Nutritional Risk Screening Tool for Preterm Infants from Birth to Corrected Age Four Months Old: A Pilot Study

**DOI:** 10.1159/000522320

**Published:** 2022-05-06

**Authors:** Xiaoying He, Zhuobin Jiang, Basma Nasr, Cuiling Wu, Saijun Huang, Pingming Gao, Yanna Zhu

**Affiliations:** ^a^Department of Maternal and Child Health, School of Public Health, Sun Yat-sen University, Guangzhou, China; ^b^Child Healthcare Department, Foshan Women and Children Hospital, Foshan, China; ^c^Information Center, Foshan Women and Children Hospital, Foshan, China; ^d^Department of Cardiology, West China Hospital of Sichuan University, Chengdu, China; ^e^Department of Neonatology, Foshan Women and Children Hospital, Foshan, China

**Keywords:** Nutritional risk, Screening tool, Preterm infant

## Abstract

**Introduction:**

Premature infants are exceptionally vulnerable to nutrition-related diseases, and the utilization of standardized feeding guidelines may reduce nutritional practice variation, which can promote growth. Nutritional risk screening is the first step for standardized nutrition advice. However, risk screening tools specific for following up preterm infants are scarce. Hence, our study aimed to develop and evaluate a standardized Nutritional Risk Screening Tool for Preterm Infants (NRSP subscale 1) from birth to corrected age four months old.

**Methods:**

This study was a two-phase (the development phase and evaluation phase) study. Initially, we used the Delphi expert consultation method to create NRSP subscale 1. Then, a professional panel interviewed the participated preterm infants using the screening tool, measured anthropometric parameters, and conducted an intellectual development test on the interview day and remeasured anthropometric parameters 2 weeks or 1 month after the first interview. In the development phase, we cross-tabulated the responses to the screening tool with the classifications of *z*-scores of the body weight, length, or head circumference to identify significant predictors of underweight, stunting, or microcephaly. We then combined significant predictors to produce models for predicting underweight, stunting, or microcephaly by multivariate logistic regression analysis. In the evaluation phase, the area under the curve (AUC), sensitivity, specificity, and correlation coefficient by Spearman's correlation analysis (*r*<sub>s</sub>) between the risk classifications by NRSP subscale 1 and the classifications of the *z*-scores of the body weight, length, or head circumference were calculated to assess the validity of the screening tool. Intellectual development levels between high and low nutritional risk infants were statistically compared.

**Results:**

A total of 219 and 244 preterm infants were included to two phases, respectively. AUC was 0.936 (95% CI: 0.860–1.000, *p* < 0.001), sensitivity was 0.667, specificity was 0.941, *r*<sub>s</sub> = 0.407 (*p* < 0.001); AUC was 0.794 (95% CI: 0.638–0.951, *p* = 0.002), sensitivity was 0.500, specificity was 0.953, *r*<sub>s</sub> = 0.339 (*p* < 0.001); AUC was 0.831 (95% CI: 0.737–0.925, *p* = 0.001), sensitivity was 0.889, specificity was 0.643, *r*<sub>s</sub> = 0.215 (*p* = 0.001) in predicting underweight, stunting, and microcephaly on the interview day, respectively. AUC was 0.905 (95% CI: 0.826–0.984, *p* = 0.006), sensitivity was 0.500, specificity was 0.905, *r*<sub>s</sub> = 0.504 (*p* < 0.001); AUC was 0.738 (95% CI: 0.515–0.960, *p* = 0.034), sensitivity was 0.429, specificity was 0.848, *r*<sub>s</sub> = 0.382 (*p* < 0.001); AUC was 0.664 (95% CI: 0.472–0.856, *p* = 0.071), sensitivity was 0.455, specificity was 0.809, *r*<sub>s</sub> = 0.169 (*p* = 0.037) in predicting underweight, stunting, and microcephaly 2 weeks or 1 month after the first interview, respectively. Gross motor development quotients (DQs) (95.85 [32.87] vs. 86.29 [17.19], *p* = 0.022), fine motor DQs (115.77 [46.03] vs. 102.12 [20.27], *p* = 0.010), and verbal DQs (110.73 [35.27] vs. 100.63 [21.28], *p* = 0.042) were higher in low nutritional risk infants than high-risk ones.

**Conclusion:**

NRSP subscale 1 was acceptable and reliable in predicting underweight, but the validity in predicting stunting or microcephaly was slightly mild. Further investigations are required to authenticate NRSP subscale 1's effectiveness.

## Introduction

Premature infants are exceptionally vulnerable to nutrition-related diseases, such as protein-energy malnutrition, iron deficiency, and metabolic bone disease [1, 2, 3, 4]. Numerous studies demonstrated that better nutrition intake correlated positively with improved growth [5, 6, 7]. Besides, the implementation of standardized feeding guidelines could minimize nutritional practice variation and facilitate growth [8].

However, standardized nutritional risk screening and nutrition advice for preterm infants upon discharge is not widely performed in China [8]. Moreover, the current nutritional risk screening tool for children aims at identifying nutritional risk for hospitalized pediatric patients [9, 10, 11], and information provided by those screening tools is not detailed enough to follow up the premature infants. Therefore, to develop a nutritional risk screening tool specific for following up the preterm infants is the first step for standardized nutrition advice [12] and of essential.

Nutrients required for catch-up growth are sufficient energy, protein, vitamins, and minerals [13, 14]. Studies also found that extrauterine growth retardation and decrease in *z*-score of body weight or length were significant predictors of growth failure in early childhood [15]. Consequently, when developing a nutritional risk screening tool specific for preterm infants, the issues mentioned above should be under consideration. The purpose of this study was to demonstrate how the nutritional risk screening tool for preterm infants from birth to corrected age four months old in outpatient settings (Nutritional Risk Screening Tool for Preterm Infants, NRSP subscale 1) is developed and the validity of the screening tool.

## Materials and Methods

### The Development of NRSP Subscale 1

Initially, a professional panel of five pediatric medical staff qualified no less than attending physicians designed the item pool. The item pool was then reviewed using the Delphi expert consultation method. Eight experts with qualifications of at least associate chief physician or associate professor specializing in pediatrics or nutriology were individually invited to assess the manuscript through email. In the first review, experts used a Likert scale (score 1 as not important at all, score 5 as very important) to evaluate each item's importance and proposed suggestions for the screening tool. We excluded items with a mean score of less than 4. The professional panel then revised the screening tool according to the experts' recommendations. Thereafter, the experts were asked to re-evaluate the validity of each item of the screening tool. Eventually, we retained items agreed by all experts only to construct NRSP subscale 1.

### Participants

This study was composed of two phases: the development phase and the evaluation phase. The development phase occurred from August to December 2020, while the evaluation phase was from January to May 2021. Participants were enrolled from the Child Healthcare Department of Foshan Women and Children Hospital by convenience sample. Recruitment of participants to both phases was based on the following inclusion and exclusion criteria. We recruited preterm infants (gestational age [GA] <37 weeks) aged from birth to corrected age four months old who undertook physical examination in the Child Healthcare Department of Foshan Women and Children Hospital. On the other hand, preterm infants who were diagnosed with metabolic diseases and required a special diet were excluded from the study. This study protocol was reviewed and approved by the Medical Ethics Committees of Foshan Women and Children Hospital, approval number FSFY-MEC-2020-028.

### Data Collection

All parents of the participating preterm infants had given their written informed consent before the interview. Five qualified healthcare workers face-to-face interviewed the caregivers using standardized guiding words and marked their choices. After the interview, the preterm infants would undergo anthropometric measurement based on standardized techniques and the intellectual development test (for preterm infants of corrected age from GA 40 weeks to 4 months) by the “Developmental Scale for Children aged 0–6 years of China.” The intellectual development level was described as the development quotient (DQ; mental age/corrected age), including gross motor index, fine motor index, adaptability index, verbal index, and social communication index. For example, for a preterm infant of corrected age 1 month whose gross motor could reach the level of term infants at 1 month, his gross motor mental age was 1 month, and his DQ was 100 (1/1). Two weeks or 1 month after the first interview, the preterm infants' anthropometrics were measured again. z-Scores of (birth) weight, length, or head circumference were calculated based on the Fenton 2013 growth chart (for preterm infants of corrected age before GA 40 weeks) or WHO growth chart (for preterm infants of corrected age from GA 40 weeks to 4 months). Clinical and anthropometric data were recorded into a spreadsheet and double-checked daily.

A subsample of 20% of participants in the development phase was interviewed on the same day by two healthcare workers independently to evaluate the inter-rater reliability. Another 20% of participants were reinterviewed after a week to evaluate the test-retest reliability. The agreement was on a reliability level of more than 80% (data not shown).

### Nutritional Risk Outcomes

The nutritional risk outcomes of NRSP subscale 1 were the classifications of *z*-score of body weight, length, and head circumference, which were measured on the interview day and 2 weeks or 1 month after the first interview. “Underweight/stunting/microcephaly at present” was defined as a *z*-score of body weight/length/head circumference on the interview day < −2. “Underweight/stunting/microcephaly next time” was described as a *z*-score of body weight/length/head circumference on the day 2 weeks or 1 month after the first interview < −2.

### Statistical Analysis

Statistical analysis was carried out using IBM SPSS, version 25.0. Normal distributed continuous data were described as mean and standard deviation values and analyzed by one-way analysis of variance or Student's *t* test or as median and interquartile range and analyzed by the Kruskal-Wallis test. At the same time, categorical data were presented as frequencies and percentages and analyzed by the χ^2^ test or Fisher's exact test.

We compared demographic data of participants of the two phases. We used data from the development phase to assign items scores and produce models to predict nutritional risk. Responses to the screening tool were cross-tabulated with the classifications of *z*-scores of body weight, length, or head circumference, respectively, by univariate analysis to identify factors that significantly predicted underweight, stunting, or microcephaly. We then assigned a score to significant factors based on their relationship with body weight, length, and head circumference growth. For example, factors with no significant relationship with underweight, stunting, or microcephaly were scored as zero (e.g., 100–150 mL/kg/day milk intake), whereas factors with a significant relationship were scored as 1 (e.g., 80–100 mL/kg/day milk intake) or 2 (e.g., <80 mL/kg/day milk intake). Factors recognized in the literature as having a direct impact on growth were scored similarly. For example, vitamin D supplement 400–800 IU/day for body length growth, “6–7 days per week” was scored as zero, “4–5 days per week” was scored as 0.5, “1–3 days per week” was scored as 1, whereas “none” was scored as 2. We then combined predictors to generate a model that would best predict underweight, stunting, or microcephaly using multivariate logistic regression analysis. The area under the curve (AUC) was used to assess the models' effectiveness in predicting underweight, stunting, or microcephaly, while the cutoff values were determined using the Youden index.

Moreover, we utilized data from the evaluation phase to accurately evaluate the validity of the screening tool. AUC, sensitivity, specificity, and correlation coefficient by Spearman's correlation analysis (*r*_s_) between risk classification by NRSP subscale 1 and the *z*-score classification of body weight, length, or head circumference will be calculated.

Finally, DQs between infants with high nutritional risk, which were estimated to have a higher risk of being underweight, stunting, or microcephaly at present, and low nutritional risk infants evaluated by NRSP subscale 1 were statistically compared. A *p* value of <0.05 was considered statistically significant.

## Results

### Demographic Data

We recruited 219 preterm infants in the development phase and 244 in the evaluation phase. All included participants were with thoroughly completed data. Our study showed no significant differences between the two samples except in the distribution of the preterm infants of different GAs (Table 1). However, *z*-scores were used as outcome indicators and were corrected with GA; thus, the aforementioned difference might not affect the statistical analysis results. We successfully measured the anthropometric parameters of 137 out of 219 and 152 out of 244 preterm infants on the day 2 weeks or 1 month after the first interview. Reasons for the absence of second anthropometric measurements included the infant being under the care of another medical center or in a seriously ill condition or moving out from Foshan. There were no significant differences between the two samples. The demographic data are shown in Table 1.

### NRSP Subscale 1

NRSP subscale 1 was established as containing four parts. Basic information included gender, GA, birthdate, weight, and height of both parents.

Part 1 primarily discussed the health status of preterm infants. It included past medical history (e.g., history of necrotizing enterocolitis, hypoxic-ischemic encephalopathy, mother diagnosed with gestational diabetes mellitus, etc.) and current diseases (e.g., gastrointestinal, cardiopulmonary, neurological and hematological diseases, metabolic and allergic disorders, and acute conditions).

Part 2 incorporated information about the feeding practices of preterm infants. For example, milk intake per kilogram of weight per day, breastfeeding exclusivity, formulae milk preparation as instructed, nutritional fortifier use (e.g., human milk fortifier, post-discharge formulae), special formulae use (e.g., extensively hydrolyzed formulae, amino acid formulae), the presence of feeding difficulty, and whether each feed duration exceeded 30 min.

Part 3 discussed the nutrients supplements such as vitamin D, vitamin A, iron element, and outdoor hours per week. Part 4 involved anthropometric assessment, including *z*-scores of (birth) weight/length/head circumference, and the presence of a descending pattern in *z*-score of body weight or length or head circumference (*z*-score on the interview day minus *z*-score last time < −0.2). Items of the NRSP subscale 1 are shown in Tables 2 and 3.

### Predictors Identified by Univariate Analysis

Our study found that birth weight and length, current diseases, milk intake per day, nutritional fortifiers and special formulae use, feeding difficulty, and the decrease in *z*-score of body weight had a significant relationship with the classification of *z*-score of body weight on the interview day. However, birth weight and length, current diseases, nutritional fortifiers use, and feeding difficulty correlated significantly with the classification of *z*-score of body length on the interview day. Meanwhile, birth head circumference, current diseases, milk intake per day, nutritional fortifiers and special formulae use, feeding difficulty, vitamin D supplement, and the decrease in *z*-score of head circumference showed correlation with the classification of *z*-score of head circumference on the interview day.

Furthermore, the analysis showed that birth weight and length, body weight and length on the interview day, current diseases, milk intake per day, nutritional fortifier and special formulae use, feeding difficulty, and iron supplement had a significant relationship with the classification of *z*-score of body weight on the day 2 weeks or 1 month after the first interview. At the same time, results exhibited a significant association between birth weight and length, body weight and length on the interview day, current diseases, and feeding difficulty with the classification of *z*-score of body length on the day 2 weeks or 1 month after the first interview. Simultaneously, birth head circumference, body weight, length and head circumference on the interview day, current diseases, milk intake per day, special formulae use, feeding difficulty, the decrease in *z*-score of head circumference related to the classification of *z*-score of head circumference on the day 2 weeks or 1 month after the first interview. Results of responses to the NRSP subscale 1 compared with the *z*-scores classification of body weight, length, and head circumference were shown in Table 2.

### Models Combined by Significant Factors to Predict Growth Retardation and Validity

After multivariate logistic regression analysis (data not shown), elements of each item were assigned scores (Table 3) by panel discussion. According to the results of univariate analysis and multivariate logistic regression analysis, the model to predict “underweight at present” included factors of *z*-scores of birth weight and length, current diseases, milk intake, nutritional fortifier use, special formulae use, feeding difficulty, and the decrease in *z*-score of body weight. The model for predicting “stunting at present” included factors of birth weight and length, current diseases, milk intake, nutritional fortifier use, feeding difficulty, vitamin D supplement, and outdoor hours per week. The model for predicting “microcephaly at present” included factors of birth head circumference, current diseases, milk intake, nutritional fortifier use, special formulae use, feeding difficulty, vitamin D supplement, and the decrease in *z*-score of head circumference.

The model for predicting “underweight next time” was composed of factors of birth weight and length, body weight and length on the interview day, current diseases, milk intake, nutritional fortifier use, special formulae use, feeding difficulty, and iron element supplement. The model for predicting “stunting next time” included factors of birth weight and length, body weight and length on the interview day, current diseases, milk intake, nutritional fortifier use, feeding difficulty, vitamin D supplement, and outdoor hours per week. The model for predicting “microcephaly next time” covered factors of birth head circumference, head circumference on the interview day, current diseases, milk intake, nutritional fortifier use, special formulae use, feeding difficulty, vitamin D supplement, and the decrease in *z*-score of head circumference.

AUCs of models to predict underweight and stunting were all above 0.700, sensitivities were 0.429–0.667, specificities were 0.848–0.953, and correlation coefficients were 0.339–0.504. AUCs of models to predict microcephaly were above 0.664, sensitivities were 0.455–0.889, specificities were 0.643–0.809, and correlation coefficients were 0.169–0.215. Results were shown in Table 4.

### Intellectual Development Levels between High and Low Nutritional Risks Preterm Infants

A total of 413 preterm infants from the development and evaluation phases underwent an intellectual development test. There was a discrepancy in the distribution of different GAs between the high and low nutritional risk groups. However, corrected age was used when calculating DQ; therefore, it was suggested that the difference mentioned above might not affect the result. DQs of low nutritional risk preterm infants were all higher than those of high nutritional risk preterm infants, but only differences of full-scale DQs (103.63 [28.04] vs. 95.59 [10.21], *p* = 0.005), gross motor DQs (95.85 [32.87] vs. 86.29 [17.19], *p* = 0.022), fine motor DQs (115.77 [46.03] vs. 102.12 [20.27], *p* = 0.010), and verbal DQs (110.73 [35.27] vs. 100.63 [21.28], *p* = 0.042) were with statistical significance. Results are shown in Table 5.

## Discussion

Detection of nutritional risk is the premise of hierarchical-targeted nutritional intervention [16]. NRSP subscale 1 developed by our research is expected to colossally assist medical staff in detecting feeding problems of preterm infants to ensure the implementation of nutritional guidelines for preterm infants.

### NRSP Subscale 1 Was Preterm-Infant-Specific

Based on the structure of the current nutritional risk screening tools [9, 17, 18, 19], NRSP subscale 1 involved factors regarding diseases, dietary intake, and anthropometric assessments. Reasons for including these factors were thoroughly evidence-based. Strong evidence indicated the presence of nutritional consequences of certain diseases in children [20]. Preterm infants discharged from neonatal intensive care units might also often suffer from cardiopulmonary, digestive, metabolic, and neurological disorders [20, 21, 22], besides acute conditions.

Human milk or formula is considered the main source of energy and protein in preterm infants corrected age <4 months [23, 24]. Additionally, nutritional fortifiers are recommended for preterm infants when necessary to prevent extrauterine growth retardation [25]. Feeding with special formulae such as extensively hydrolyzed formulae might also affect the growth of preterm infants [26, 27]. Feeding difficulty might lead to insufficient intake and waste of massive energy [28, 29]. Furthermore, preterm infants usually suffer from nutrients (e.g., vitamin A, D, iron) insufficiency [2, 3]. Ultimately birth weight, a decrease in *z*-score for body weight or length is considered a predictor of growth retardation; at the same time, very low birth weight might be a risk factor for the subsequent occurrence of growth hormone deficiency [15, 30].

Therefore, we should thoroughly consider all issues mentioned above when conducting nutritional risk screening for preterm infants. During the development of NRSP subscale 1, the professional panel and experts elaborately modified the items which covered issues aforementioned and diligently organized the structure to ensure that the screening tool was comprehensive and detailed yet not time-consuming. By designing NRSP subscale 1, we intended to ease healthcare workers conducting a standardized nutritional risk screening, thus providing more targeted advice.

### The Validity of NRSP Subscale 1 Was Relatively Reliable

Results from the pilot study demonstrated that the newly developed screening tool might be reliable. The screening tool's AUCs in predicting both underweight and stunting were 0.738–0.936, which suggested the accuracy of the prediction was moderate to high [31]; however, the AUCs in predicting microcephaly were 0.664–0.831, which were slightly lower than those of underweight and stunting.

Sensitivities of NRSP subscale 1 were 0.429–0.889, which were slightly lower than those of the Paediatric Youkhill Malnutrition Score (PYMS, 59%) [19] and the Screening Tool for the Assessment of Malnutrition in Paediatrics (STAMP, 70%) [18]. Meanwhile, specificities were 0.643–0.953, which were also lower than those of PYMS (92%) [19] and STAMP (91%) [18].

We also found that NRSP subscale 1 was more effective in predicting underweight (*r*_s_ 0.407–0.504) than stunting or microcephaly (*r*_s_ < 0.4). That was because the body length or head circumference growth was regulated by genetic factors, social and economic environment, nutrition, health status, cerebral development, and skull thickness [32, 33, 34]. Although the correlation between the *z*-scores classifications of body weight, length, or head circumference and the nutritional risk classification by NRSP subscale 1 was relatively low, we could still benefit from the screening tool. As genetic factors, social and economic environments are not easy to change; however, improving health status or nutrition intake is feasible.

Furthermore, we found that the intellectual development indexes of low nutritional risk preterm infants were higher than those of high nutritional risk ones, which also indicated that the nutritional risk classification by NRSP subscale 1 was effective. The result suggested that healthcare staff should shed light on improving the feeding practice of preterm infants with high nutritional risk to facilitate their intellectual development.

This study's main limitations were that this study was a pilot study in design and the relatively small sample size. However, the sample size of each phase was more than five times the amount of the screening tools' items [35]; thus, it was acceptably enough for the preliminary development of NRSP subscale 1. Also, taking the relatively low sensitivity and specificity of the screening tool into consideration, we still need a large-scale multicenter study to broadly promote the models of NRSP subscale 1. That will be the next step in our protocol.

## Conclusion

The present study described the steps for developing and evaluating a nutritional risk screening tool for preterm infants aged from birth to corrected age four months old. The newly developed tool was preterm-infant-specific, covering their most common issues (e.g., diseases spectrum, nutrients supplement). We found the screening tool relatively reliable. Moreover, the screening tool's questions and guiding words were elaborately modified by a professional panel and experts. We expect it aiding in standardizing the process of nutritional risk screening in preterm infants' follow-up. Nevertheless, further investigations are required to establish the screening tool's effectiveness within different healthcare settings, such as community health services.

## Statement of Ethics

This study protocol was reviewed and approved by the Medical Ethics Committees of Foshan Women and Children Hospital, approval number FSFY-MEC-2020-028. All parents of the participating preterm infants had given their written informed consent before the interview.

## Conflict of Interest Statement

The authors have no conflicts of interest to declare.

## Funding Sources

This work was supported by Guangdong Medical Science and Technology Research Fund Project, grant number A2020472.

## Author Contributions

X.H. and Y.Z. proposed and designed the study. X.H., C.W., S.H., P.G., and Y.Z. drafted the item pool. X.H., C.W., and S.H. carried out the study. X.H. and Z.J. performed the statistical analysis and were co-first authors. X.H., Z.J., and B.N. drafted and revised the manuscript. All the authors read and approved the final manuscript.

## Data Availability Statement

All data generated or analyzed during this study are included in this article. Further inquiries can be directed to the corresponding author.

## Figures and Tables

**Table 1:** Demographic data of recruited participants

Phase	Development	Evaluation	*p* value
*Data of the interview day*			
Total	219	244	
Male, *n* (%)	118 (53.9)	143 (58.6)	0.306*
Gestation age, *n* (%)			
<32 weeks	42 (19.2)	72 (29.5)	
32–34 weeks	69 (31.5)	55 (22.5)	0.014*
>34 weeks	108 (49.3)	117 (48.0)	
*z*-Score of birth weight, M (IQR)	−0.26 (−0.82, 0.33)	−0.32 (−0.79, 0.22)	0.549^#^
*z*-Score of birth length, M (IQR)	−0.12 (−0.87, 0.20)	−0.30 (−0.76, 0.36)	0.917^#^
*z*-Score of birth head circumference, M (IQR)	−0.38 (−1.23, 0.24)	−0.56 (−1.06, 0.06)	0.660^#^
Corrected age, months, M (IQR)	0.63 (0.07, 2.13)	0.90 (0.13, 2.17)	0.420^#^
*z*-Score of body weight, M (IQR)	−0.05 (−0.80, 0.71)	0.02 (−0.69, 0.67)	0.192^#^
*z*-Score of body length, M (IQR)	−0.14 (−0.84, 0.65)	−0.16 (−0.73, 0.42)	0.679^#^
*z*-Score of head circumference, M (IQR)	0.38 (−0.56, 1.05)	0.66 (−0.06, 1.21)	0.071^#^

*Data of the day 2 weeks or 1 month after the first interview*			
Total	137	152	
Male, *n* (%)	81 (59.1)	93 (61.2)	0.721 *
Gestation age, *n* (%)			
<32 weeks	31 (22.6)	48 (31.6)	0.069*
32–34 weeks	50 (36.5)	38 (25.0)	
>34 weeks	56 (40.9)	66 (43.4)	
*z*-Score of birth weight, M (IQR)	−0.32 (−0.97, 0.14)	−0.34 (−0.77, 0.16)	0.855^#^
*z*-Score of birth length, M (IQR)	−0.28 (−1.04, 0.08)	−0.32 (−0.77, 0.30)	0.406^#^
*z*-Score of birth head circumference, M (IQR)	−0.54 (−1.27, 0.20)	−0.56 (−1.06, 0.06)	0.951^#^
Corrected age, months, M (IQR)	1.83 (0.39, 2.77)	1.86 (0.80, 3.31)	0.164^#^
*z*-Score of body weight, M (IQR)	0.14 (−0.58, 0.82)	0.34 (−0.39, 0.86)	0.591^#^
*z*-Score of body length, M (IQR)	−0.01 (−0.89, 0.78)	−0.06 (−0.76, 0.67)	0.589^#^
*z*-Score of head circumference, M (IQR)	0.62 (−0.18, 1.20)	0.80 (−0.01, 1.60)	0.051^#^

M, median; IQR, interquartile range.

* Using χ^2^ test.

# Using the Kruskal-Wallis test.

**Table 2:** Relationship of responses to the screening tool with the *z*-scores classification of body weight, length, and head circumference by univariate analysis

	WTZ1	HTZ1	HCZ1	WTZ2	HTZ2	HCZ2
Father height*	−	−	−	−	−	−
Father weight*	−	−	−	−	−	−
Mother height*	−	−	−	−	−	−
Mother weight*	−	−	−	−	−	−
Past medical history^#^	−	−	−	−	−	−
Current diseases^#^	+	+	+	+	+	+
Milk intake per day^#^	+	−	+	+	−	+
Breastfeeding exclusivity^#^	−	−	−	−	−	−
Formulae preparation as instructed^#^	−	−	−	−	−	−
Nutritional fortifier use^#^	+	+	+	+	−	−
Special formulae use^#^	+	−	+	+	−	+
Feeding difficulty^#^	+	+	+	+	+	+
Duration of each feeding^#^	−	−	−	−	−	−
Vitamin D supplement^#^	−	−	+	−	−	−
Outdoor hours per week^#^	−	−	−	−	−	−
Iron element supplement^#^	−	−	−	+	−	−
Vitamin A supplement^#^	−	−	−	−	−	−
*z*-Score of birth weight^#^	+	+	−	+	+	−
*z*-Score of birth height^#^	+	+	−	+	+	−
*z*-Score of birth head circumference^#^	−	−	+	−	−	+
Decrease in *z*-score of body weight*	+	−	−	−	−	−
Decrease in *z*-score of body height^#^	−	−	−	−	−	−
Decrease in *z*-score of head circumference^^#^^	−	−	+	−	−	+
WTZ1^#^				+	+	+
HTZ1^#^				+	+	+
HCZ1^#^				−	−	+

WT/HT/HCZ1, *z*-scores of body weight/length/head circumference on the interview day; WT/HT/HCZ2, *z*-scores of body weight/length/head circumference on the day 2 weeks or 1 month after the first interview.

* Using one-way analysis of variance.

# Using the χ^2^ test.

*p* < 0.05 in univariate analysis.

^−^ *p* > 0.05 in univariate analysis.

**Table 3:** Scores of each item of nutritional risk screening tool for preterm infants

Items
1. Health status

1.1. Past medical history A: none (score 0), B: serious gastrointestinal disorders/cardiopulmonary diseases or invasive operation/neurological disorders/metabolii disorders/hematological system diseases/infectious diseases (score 2), and C: maternal diseases during pregnancy (score 1)

1.2. Current diseases A: none (score 0), B: gastrointestinal disorders/cardiopulmonary disorders/neurological disorders/metabolic disorders/hematological system diseases with functional impairment (score 2), and C: acute diseases/allergic diseases (score 1)

2. Feeding practice

2.1. Milk intake per kilogram of weight per 24 hours A: <80 mL/kg/day (score 2), B: 80–100 mL/kg/day (score 1), and C: 100–150 mL/kg/day (score 0)

2.2. Breastfeeding exclusivity^†^ A: all, B: partial, and C: none

2.3. Formulae preparation as instructed A: yes (score 0) and B: no (score 1)

2.4. Nutritional fortifier use A: more than half of the milk intake per day (score −2), B: less than half of the milk intake per day (score −1), and C: none (score 0)

2.5. Special formulae use A: more than half of the milk intake per day (score 2), B: less than half of the milk intake per day (score 1), and C: none (score 0)

2.6. Duration of each feeding^†^ A: >30 min and B: ≤30 min

2.7. Feeding difficulty A: easy (score 0), B: difficult (score 1), and C: very difficult (score 2)

3. Nutrients supplement

3.1. Supply vitamin D 400–800 IU as the daily dose A: 6–7 days per week (score 0), B: 4–5 days per week (score 0.5), C: 1–3 days per week (score 1), and D: none (score 2)

3.2. Outdoor hours per week A: more than 7 h per week (score −1), B: 5–7 h per week (score 0), C: 3–5 h per week (score 0.5), D: 1–3 h per week (score 1), and E: less than 1 h per week (score 2)

3.3. Supply vitamin A 1,333–1,500 IU as the daily dose A: 6–7 days per week (score 0), B: 4–5 days per week (score 0.5), C: 1–3 days per week (score 1), and D: none (score 2)

3.4. Supply iron element 2 mg per kilogram of weight as the daily dose A: 6–7 days per week (score 0), B: 4–5 days per week (score 0.5), C: 1–3 days per week (score 1), and D: none (score 2)

3.5. Supply other nutrients such as calcium, multivitamin, DHA, prebiotics^†^ A: none and B: yes

4. Anthropometric assessment

4.1. z–Score of birth weight A: ≥ −1 (score 0), B: − 1~ −2 (score 1), and C: < −2 (score 2)

4.2. *z*-Score of birth length A: ≥ −1 (score 0), B: − 1~ −2 (score 1), and C: < −2 (score 2)

4.3. *z*-Score of birth head circumference A: ≥ −1 (score 0), B: −1~ −2 (score 1), and C: < −2 (score 2)

4.4. Decrease in *z*-score of body weight (*z*-score on the interview day minus *z*-score last time < −0.2) A: No (score 0) and B: yes (score 2)

4.5. Decrease in *z*-score of body length A: No (score 0) and B: yes (score 2)

4.6. Decrease in *z*-score of head circumference A: No (score 0) and B: yes (score 2)

4.7. *z*-Score of body weight on the interview day A: ≥ −1 (score 0), B: −1~ −2 (score 1), and C: < −2 (score 2)

4.8. *z*-Score of body length on the interview day A: ≥ −1 (score 0), B: −1~ −2 (score 1), and C: < −2 (score 2)

4.9. *z*-Score of head circumference on the interview day A: ≥ −1 (score 0), B: −1~ −2 (score 1), and C: < −2 (score 2)

† Items that were not included in models to predict nutritional risk were not assigned with the score.

**Table 4:** The validity of nutritional risk screening tool for preterm infants

Nutritional risk	AUC1 (95% CI)	AUC2 (95% CI)	Sensitivity	Specificity	*r_s_*
Underweight at present	0.920 (0.805–1.000)*	0.936 (0.860–1.000)*	0.667	0.941	0.407*
Stunting at present	0.892 (0.733–1.000)*	0.794 (0.638–0.951)*	0.500	0.953	0.339*
Microcephaly at present	0.848 (0.600–1.000)*	0.831 (0.737–0.925)*	0.889	0.643	0.215*
Underweight next time	0.955 (0.873–1.000)*	0.905 (0.826–0.984)*	0.500	0.905	0.504*
Stunting next time	0.978 (0.953–1.000)*	0.738 (0.515–0.960)*	0.429	0.848	0.382*
Microcephaly next time	0.675 (0.523–0.828)*	0.664 (0.472–0.856)^#^	0.455	0.809	0.169*

AUC1, area under the curve of the development phase; AUC2, area under the curve of the evaluation phase; *r*_s_, correlation coefficient of Spearman's correlation analysis.

* < 0.05.

# *p* < 0.01.

**Table 5:** Intellectual development quotients between high and low nutritional risk preterm infants (mean [SD])

	High nutritional risk	Low nutritional risk	*p* value
*n*	50	363	
Male, *n* (%)	28 (56.00)	205 (56.43)	0.942^#^
GA, week, M (IQR)	35.43 (33.71, 36.57)	34.72 (32.71, 36.00)	0.012^†^
Gross motor DQs	86.29 (17.19)	95.85 (32.87)	0.022*
Fine motor DQs	102.12 (20.27)	115.77 (46.03)	0.010*
Adaptability DQs	91.67 (28.05)	99.33 (41.19)	0.221*
Verbal DQs	100.63 (21.28)	110.73 (35.27)	0.042*
Social communication DQs	95.87 (18.27)	100.55 (26.50)	0.249*
Full-scale DQs	95.59 (10.21)	103.63 (28.04)	0.005*

DQ, development quotient; M, median; IQR, interquartile range; SD, standard deviation.

# Using the χ^2^ test.

† Using the Kruskal-Wallis test.

* Using the Student *t* test.

## Notes

Xiaoying He and Zhuobin Jiang were co-first authors of this study.
